# Oral Hygiene Facilitators and Barriers in Greek 10 Years Old Schoolchildren

**DOI:** 10.5005/jp-journals-10005-1290

**Published:** 2015-08-11

**Authors:** Matina Angelopoulou, Katerina Kavvadia, Constantine Oulis, Christina Reppa

**Affiliations:** Assistant Professor, Department of Developmental Sciences, Marquette University School of Dentistry, WI, United States; Associate Professor, Department of Pediatric Dentistry, Dental School, University of Athens, Attica, Greece; Professor, Department of Pediatric Dentistry, Dental School, University of Athens, Attica, Greece; Postgraduate Student, Department of Pediatric Dentistry, Dental School, University of Athens, Attica, Greece

**Keywords:** Barriers, Facilitators, Motivation, Oral hygiene, Schoolchildren, Toothbrushing.

## Abstract

**Aim:** The aim of this study was to determine the oral hygiene facilitators and barriers for 10 years old Greek children, via a questionnaire and clinical examination.

**Materials and methods:** This was a cross-sectional study of 266, 10 years old, children recruited from schools in 3 locations in Greece. Data were collected via questionnaires and clinical examination. Questionnaires referred to Children’s oral hygiene knowledge, behavior and attitude as well as parents’ oral hygiene behavior and educational level. Children were clinically examined by two calibrated pediatric dentists using a WHO probe and artificial light to assess dental plaque (hygiene index-HI), gingivitis (simplified gingival index-GIs) and dental caries (DMFT-BASCD criteria).

**Results:** Regarding oral hygiene knowledge, although 80% of the children were literate of the proper means of oral hygiene, only 58.64% brushed their teeth twice daily and 36.84% used dental floss. Children’s oral hygiene knowledge was positively correlated with both parental brushing frequency (ρ = 0.175, p < 0.05) and educational level (ρ = -0.216, p < 0.05). Toothpaste use was reported by 92.11% of the children. Regarding Children’s attitude, 62.28% were concerned whether their teeth were clean, with girls showing greater concern than boys (p < 0.001). Their reported beliefs regarding brushing avoidance were boredom (84.06%), low oral health literacy (73.91%) and forgetfulness (56.52%).

**Conclusion:** Oral hygiene facilitators were found to be the concern about how clean were their teeth, oral health literacy of both children and parents and toothpaste appeal to children. Oral hygiene barriers were Children’s boredom, low oral health literacy, forgetfulness and low socioeconomic level.

**How to cite this article:** Angelopoulou M, Kavvadia K, Oulis C, Reppa C. Oral Hygiene Facilitators and Barriers in Greek 10 Years Old Schoolchildren. Int J Clin Pediatr Dent 2015;8(2):87-93.

## INTRODUCTION

Oral hygiene is the most effective measure to prevent caries and periodontal disease.^1,2^ Ideally brushing should be performed twice a day in order to maintain oral health.^1^ However, many children globally brush less than once a day.^3-5^ More specifically in Greece, results of a recent epidemiological survey showed that 68.7% of 12 years old children brush occasionally, 78,2% had average or poor oral hygiene while 41.5% had gingivitis.^6^ The above findings show a necessity to define the facilitators and barriers of oral hygiene in order to motivate children and improve their oral health.

Motives for oral hygiene have been examined in the past in adolescents.^7,8^ Results of these studies suggest that concerns of teeth cleanliness, attraction to the opposite gender, self-esteem and family structure can facilitate or impede the performance of oral hygiene in adolescents.^7,8^ The influence of socioeconomic factors on oral hygiene practices among primary schoolchildren have been extensively studied.^5,9-12^ Facilitators of oral hygiene in primary schoolchildren found previously were high self-esteem, peers influence and personal appearance.^13-16^ However, clinical oral health status of children has not reported in any of the previous studies regardless the fact that is a more objective method to evaluate oral health rather than questionnaires or interviews. Also, the target group in these studies was greater than 10 years old and no data exist in younger children. Moreover, most studies focus on specific factors influencing the oral hygiene and do not investigate the variety of facilitators and barriers in primary schoolchildren.

The aim of this study was to determine facilitators and barriers of oral hygiene in 10 years old Greek school-children in relation to socioeconomic data, Children’s oral hygiene knowledge, behavior, attitude and clinical oral status.

## MATERIALS AND METHODS

### Study Design

This was a cross-sectional study of facilitators and barriers of oral hygiene in primary schoolchildren in relation to socioeconomic data, Children’s oral hygiene knowledge, behavior, and attitude and clinical oral status. After parental informed consent oral hygiene knowledge, behavior and attitude of children and their parents were evaluated via questionnaire while clinical parameters were evaluated through clinical examination. This study has been conducted in full accordance with the World Medical Association Declaration of Helsinki and was approved by the Athens University Ethical Committee and the Greek Ministry of Education (30.10.09, No126516/ Г7).

### Outcome Measures

The primary outcome was to evaluate the level of oral hygiene, knowledge, behavior, attitude, clinical oral status and socioeconomic level in order to determine the facilitators and barriers of oral hygiene.

### Sample

Inclusion criteria for sample recruitment was based on: (a) the age to be attending the 4th grade of primary school (9 to 10 years old), (b) the type of population to be of rural, low urban or high urban locations, as determined by the Hellenic Statistical Authority, (c) the schools to be from the ones participated in the national oral health education program and (d) the children not to have contributory medical history.

The sample recruited, consisted of 266, 10 years old students from seven public primary schools around Greece.

### Questionnaires

After parental informed consent, data regarding Children’s oral hygiene knowledge, attitude and behavior were collected via a questionnaire, completed at the school. The questionnaire had multiple choice questions on knowledge of toothbrushing, dental flossing, oral health behavior, parental involvement in oral hygiene and Children’s feelings about oral cleanliness and barriers that lead to brushing avoidance. A different questionnaire was send to the parents regarding their educational level and brushing frequency. Both questionnaires were distributed to 20 persons prior to their application for validation.

### Clinical Examination

All children were clinically examined in their classroom by two calibrated pediatric dentists, under all infection control measures, using a mirror, a periodontal probe (WHO-621) and artificial light. The following variables were recorded: (a) dental plaque by a modification of hygiene index (HI) of Lindhe, without the use of a disclosing agent 18, (b) gingivitis as presence or absence of gingival bleeding upon periodontal probing (WHO periodontal probe) by the simplified gingival index (GI-S)^17^ and (c) dental caries (DMFT), according to the diagnostic criteria of the British Association of Community Dentistry, BASCD.^18^

### Statistical Analysis

Power analysis was performed with G* Power software and was 86% at α = 0.05.

Data were reported descriptively by calculating Frequency, Mean and Standard Deviation (SD). For caries index, inter examiner reliability was assessed using the intraclass correlation coefficient (ICC) in 20 patients.

Spearman’s correlation coefficient was used to investigate any correlation between the various parameters from the questionnaire and clinical examination. Mann-Whitney U-test/Kruskal-Wallis test were used for statistical comparison between demographic data and data from the questionnaires and clinical examination. Nonparametric tests were used since data did not have a normal distribution. Statistical significant differences were investigated at the level of p < 0.05 using SPSS software (IBM SPSS Statistics 20.0 Armonk, NY).

## RESULTS

Sample demographic data and level of parental education are presented in Table 1.

### Oral Hygiene Knowledge

Regarding brushing frequency, 82.71% of children knew that they should brush their teeth at least twice a day. Children with knowledge of appropriate brushing frequency brushed more frequently (ρ = 0.288, p < 0.001), had lower Hygiene Index (ρ = 0.186, p < 0.05), were most often girls (ρ = 0.185, p < 0.05) and their parents brushed as well more frequently (ρ = 0.175, p < 0.05). Correlation between the variables are presented in Table 2.

Regarding dental floss’s use, 77.44% knew that it is used to clean the interproximal surfaces of teeth. Children with knowledge of appropriate use of the dental floss used it more frequently (ρ = 0.260, p < 0.001) and their parents had higher educational level (p < 0.05).

### Oral hygiene Behavior

**Graph 1:**
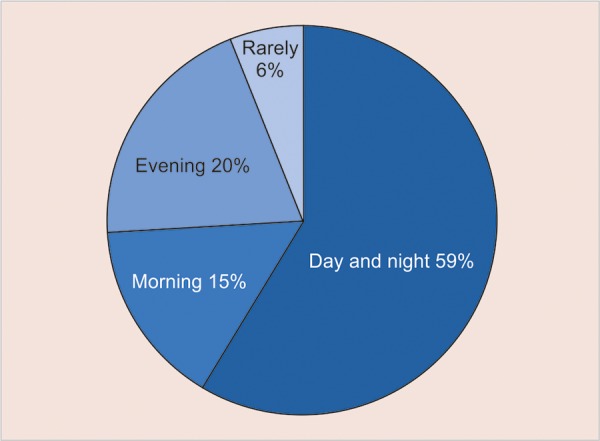
Brushing frequency in 10 years old children

Regarding brushing frequency, as presented in the pie chart in Graph 1, 58.64% reported that they brush their teeth at least twice a day. Toothpaste use was reported by 92.11% of the children and flossing by 36.84%.

**Table 1:** **Table 1:** Demographic data of the sample

				*N*	
*Children*		*Gender*			
		Male		142	
		Female		124	
		*Type of population*			
		Low urban		89	
		Rural		98	
		High urban		79	
*Parentr*		*Father’s educational level*			
		Low		50	
		Moderate		86	
		Higher		122	
		Unknown		8	
		*Mother’s educational level*			
		Low		46	
		Moderate		74	
		Higher		138	
		Unknown		8	
		Total		266	

Those children that had their parents involved during brushing, used flossing more often (ρ = 0.349, p < 0.001). Also, lower dmft score was associated with higher flossing frequency (p < 0.05).

### Oral Hygiene Attitude

Concerning their attitude, 61.28% were very concerned about how clean were their teeth with girls being significantly more concerned than boys (p < 0.001).

**Graph 2:**
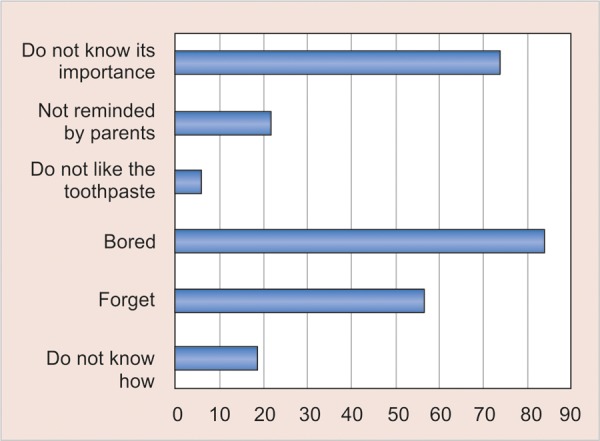
Histogram of children beliefs’ regarding brushing avoidance of their peers

Brushing avoidance for this age group and Children’s own beliefs’ are presented in the histogram of Graph 2.

### Oral Status

Regarding the sample’s clinical parameters, DMFT was 0.65 (SD = 1.15), dmft was 1.74 (SD = 2.53), hygiene index was 57.40% (SD = 29.26) and Gingival Index was 33.60% (SD = 20.64). Inter examiners reliability for dmf t/DMFT index was ICC = 0.89. Caries index of primary dentition (dmft) was correlated with parental educational level (ρ = -0.305, p < 0.001), the lower the educational level the higher the dmft index (p < 0.001). Children with higher hygiene index had significantly lower caries (ρ = -0.166, p < 0.05) and less gingivitis (ρ= -0.608, p < 0.001).

## DISCUSSION

Results of the present study suggest that facilitators for oral hygiene in 10-year-old Greek schoolchildren are Children’s and parents’ oral health education, the appeal to the toothpaste and the concern for oral cleanliness (Table 3). Barriers are the low socioeconomic level, boredom, poor oral hygiene literacy and forgetfulness (Table 3).

The sample of the present study was selected from participating schools in the Greek national oral health education program.^19^ The sample was chosen so as to include schools from rural and urban areas of low and high socioeconomic levels.

The specific age group was selected because children at that age can express their own beliefs without the need of parental involvement. Also, children at this age are capable of expressing their opinion more accurately than when their parents answer on their behalf.^20^ Moreover, a recent epidemiological study in Greece showed that 12 years old children had high prevalence of gingivitis.^6^ This finding suggested that oral health education should be implemented at an earlier age in order to improve plaque removal and control gingivitis, later in adolescence. As reported oral health habits formed in early years can lead to healthy habits during adolescence and adulthood.^13,21^

The oral status was assessed through quantitative evaluation using a questionnaire and clinical examination trying to present more accurate results using an objective evaluation method.^22^ The clinical indexes of hygiene and gingivitis were chosen because they are simple, easy and have high reliability, since they are based on the presence or absence of bleeding and dental plaque despite examiner’s estimation.^17^ Dental caries were assessed using the commonly used BASCD criteria and the DMFT index so as to compare the results with previous studies and epidemiological data.

**Table 2:** **Table 2:** Spearman’s correlation coefficient between demographic characteristics, data from the questionnaires and clinical examination

*Spearman’s* ρ*and p-value*																										
		*Demographic*				*Questionnaire*														*Clinical examination*						
*Variables*		*Gender*		*Educational Level*		*Parents’ brushing frequency*		*Brushing frequency knowledge*		*Flos use knowledge*		*Brushing frequency behaviour*		*Flos use behaviour*		*Parental involvement in brushing*		*Oral Clenliness concern*		*dmft*		*DMFT*		*HI*		*GI-s*
Educational level		0.089																								
		0.322																								
Parents’ brushing frequency		0.132		0.112																						
		0.130		0.954																						
Brushing frequency knowledge		0.185		-0.134		0.175																				
		0.003*		0.139		0.048*																				
Floss use knowledge		-0.044		-0.216		0.033		-0.098																		
0.480		0.480		0.014*		0.703		0.113																		
Brushing frequency behavior		0.083		0.113		-0.034		0.288		-0.133																
		0.178		0.210		0.701		0.000*		0.030*																
Floss use behavior		0.010		0.037		-0.051		-0.154		0.260		-0.055														
		0.873		0.679		0.559		0.013*		0.000*		0.376														
Parental involvement in brushing		-0.070		0.017		0.065		-0.097		0.100		0.025		0.349												
		0.178		0.847		0.461		0.121		0.105		0.683		0.000*												
Oral cleanliness concern		0.197		-0.010		-0.094		-0.060		-0.008		-0.071		0.096		0.028										
		0.001*		0.911		0.283		0.341		0.895		0.249		0.120		0.658										
dmft		-0.102		-0.383		0.081		0.039		0.016		-0.065		-0.161		-0.010		-0.079								
		0.170		0.000*		0.400		0.609		0.834		0.380		0.029*		0.889		0.292								
DMFT		0.078		-0.148		-0.022		-0.004		-0.066		0.086		-0.089		-0.041		-0.089		0.335						
		0.297		0.098		0.816		0.953		0.375		0.245		0.234		0.585		0.234		0.000*						
HI		0.020		0.092		0.104		0.186		-0.121		0.127		-0.082		-0.082		0.048		-0.166		-0.128				
		0.790		0.306		0.280		0.012*		0.268		0.086		0.268		0.271		0.522		0.024*		0.084				
Gl-s		0.103		-0.022		-0.162		-0.109		0.138		-0.028		0.138		0.047		-0.051		0.036		0.122		-0.608		
		0.168		0.808		0.091		0.148		0.063		0.702		0.063		0523		0.496		0.624		0.100		0.000*		

*p < 0.05

**Table 3:** **Table 3:** Facilitators and barriers for oral hygiene in 10 years old children

*Facilitatorr*		*Barrierr*	
Enhancement of oral health knowledge through oral health education		Lower socioeconomic level is related to poorer oral health	
Improve parental oral health behavior through oral health education		Common reason for brushing avoidance is boredom	
The use of appealing toothpaste as a motive for oral health behavior improvement		Literate the schoolchildren regarding the importance of oral hygiene in order to apply it	
Children concerns about oral cleanliness as a motive to enhance their oral hygiene		Children tend to forget to brush their teeth and need reminders	

Data from this study show that although schoolchildren knew the appropriate brushing frequency of twice a day^1-3^ still 30% does not practice it, showing that knowledge is not adequate to adopt a brushing habit.^13,23,24^ Knowledge of brushing frequency was correlated with actual reported frequency of brushing and oral hygiene clinical findings, showing that children that have the knowledge tend to brush more frequently. Thus, as has been reported in the past. Daily tooth brushing frequency of children found presently was similar to the one reported in other studies.^3-5,11,15,25^ Parental brushing frequency as expected was correlated with knowledge of brushing frequency of children.^9,10,12,26,27^ The above shows that children follow the parental behavior and thus parents should also be the target in the oral health education programs addressed to children.^16,28^ However, peers influence has also been reported in young schoolchildren.^14^

Toothpaste is much enjoyed and is widely used by schoolchildren, suggesting that making the toothpaste more appealing can be a valuable tool in caries reduction and in this age population.^29,30^ Thus, toothpaste industry could focus more in offering appealing flavors and attractive designs with adequate fluoride in schoolChildren’s toothpastes. Also, oral health promotion programs should direct their efforts on free distribution of toothpastes to children.

Many children knew the use of the dental floss and this was positively correlated with parental educational level. This finding maybe attributed to the rarer use of dental floss by parents with lower educational level so this behavior was not known from children.^1,5,10,11,15^ Practicing flossing by children was positively correlated with supervised brushing by their parents showing that parents with oral hygiene literacy understand better the importance of their Children’s oral hygiene. However, knowledge is not sufficient to persuade their children to floss their teeth since only 37% of children reported the use of floss for their oral hygiene. Limited use of floss has been reported in the past.^3,15,31^ Primary schoolChildren’s attitude regarding oral hygiene has been limitedly reported in dental literature. In the current study it is apparent that mouth cleanliness and personal appearance is of high importance.^1,14,16,23^ This finding was more evident for girls, possibly because girls of that age are more mature than boys, entering their adolescence when personal appearance is quite important. Besides, many studies have reported that girls tend to have better oral hygiene.^3,5,7,9,11,15,25^

Barriers found for the application of oral hygiene in schoolchildren were boredom, ignorance of oral hygiene’s importance and forgetfulness. Forgetfulness and lack of time have been previously reported as oral hygiene barriers in older children.^13^ These findings can help oral health education programs to set realistic goals. It is well known that oral hygiene can be improved with oral health education.^32-34^ Especially if it is implemented in regular intervals to sustain this knowledge^32-36^ and possibly including more powerful scientific evidence regarding oral hygiene negligence outcomes.^28^ More importantly dental providers should focus on methods to remind and motivate children to oral hygiene.^28^ Oral hygiene campaigns using schoolChildren’s favorite athletes, movie stars or singers may help improve their oral health. Stickers, posters, TV commercials and phone apps are some other means to be included.^1,23^ Clocks and timers that remind children to brush their teeth could also be useful,^16^ as well as keeping the toothbrush next to the child’s bed to remind them to brush and to motivate them to do it without making the effort to visit the bathroom.^28^

Results of this study could be used in the future for the design of oral health education programs for this specific age group. Also, it would be interesting to develop similar studies in other cultures do define the facilitators and barriers in other cultures. Moreover, since barriers have been defined, governments, oral health companies and dental professional should focus on taking measures to overcome these barriers.

Concerns about how clean were their teeth, oral health literacy of Children’s and parents’ and their choice of toothpaste were found as facilitators for oral hygiene, whereas, Children’s boredom, low oral health literacy, forgetfulness and low socioeconomic level were found as barriers.
